# Diversity of *Mycobacterium tuberculosis* and drug resistance in different provinces of Papua New Guinea

**DOI:** 10.1186/s12866-014-0307-2

**Published:** 2014-12-05

**Authors:** Serej D Ley, Paul Harino, Kilagi Vanuga, Ruben Kamus, Robyn Carter, Christopher Coulter, Sushil Pandey, Julia Feldmann, Marie Ballif, Peter M Siba, Suparat Phuanukoonnon, Sebastien Gagneux, Hans-Peter Beck

**Affiliations:** Swiss Tropical and Public Health Institute, Basel, Switzerland; University of Basel, Basel, Switzerland; Papua New Guinea Institute of Medical Research, Goroka, Papua New Guinea; Goroka Provincial Hospital, Goroka, Papua New Guinea; Alotau Provincial Hospital, Alotau, Papua New Guinea; Queensland Mycobacterium Reference Laboratory, Pathology Queensland, Brisbane, Australia

**Keywords:** Tuberculosis, Papua New Guinea, Genotyping

## Abstract

**Background:**

Papua New Guinea (PNG) is a high tuberculosis (TB) burden country of the WHO Western Pacific Region, but so far research on drug resistance (DR) and genotypes of *Mycobacterium tuberculosis (M. tuberculosis*) was only conducted in few provinces in the country. The aim of the present study was to obtain baseline data on the level of drug resistance and the genotypic diversity of circulating *M. tuberculosis* in additional provinces and to investigate the differences between three selected sites across PNG.

**Results:**

Genotyping of 147 *M. tuberculosis* clinical isolates collected in Goroka, Eastern Highlands Province, in Alotau, Milne Bay Province and in Madang, Madang Province revealed three main lineages of *M. tuberculosis:* Lineage 4 (European-American lineage), Lineage 2 (East-Asian lineage) and Lineage 1 (Indo-Oceanic lineage). All three lineages were detected in all three sites, but the individual lineage compositions varied significantly between sites. In Madang Lineage 4 was the most prevalent lineage (76.6%), whereas in Goroka and Alotau Lineage 2 was dominating (60.5% and 84.4%, respectively) (p < 0.001). Overall, phenotypic drug susceptibility testing showed 10.8% resistance to at least one of the first-line drugs tested. Of all resistant strains (23/212) 30.4% were Streptomycin mono-resistant, 17.4% were Isoniazid mono-resistant and 13% were Rifampicin mono-resistant. Multi-drug resistant (MDR) TB was found in 2.8% of all tested cases (6/212). The highest amount of MDR TB was found in Alotau in Milne Bay Province (4.6%).

**Conclusion:**

A large number of drug resistant TB infections are present in the country and MDR TB has already been detected in all three surveyed regions of PNG, highlighting the importance of monitoring drug resistance and making it a high priority for the National Control Program. Due to the high prevalence of Lineage 2 in Milne Bay Province and given the frequent association of this lineage with drug resistance, monitoring of the latter should especially be scaled up in that province.

## Background

Over the last decade, evidence for the impact of the bacterial genetic background on TB infection and disease has strongly increased [1]. A phylogeography of *M. tuberculosis* based on large sequence polymorphisms and confirmed by multi-locus sequence analysis could furthermore be established, showing an association between specific *M. tuberculosis* strains and a particular geographic region [2,3]. The different *M. tuberculosis* strains were grouped into 6 main lineages (a 7th was recently added [4]). Various studies investigated the impact of different MTBC lineages on the clinical presentation of the disease. Infection with Lineage 2 has for example been found to be associated with faster progression to disease [5] and drug resistance [6,7]. Lineage 4 on the other hand, has been associated with pulmonary TB rather than extrapulmonary TB [8]. In addition, few studies have analysed the impact of the genetic background of both, humans and bacteria on disease development [8,9] and found correlations between different human genetic polymorphisms with specific *M. tuberculosis* lineages. However, findings from studies investigating lineage specific associations have also reflected within lineage variability [10,11]. Therefore, further discriminatory strain differentiation methods should be considered when investigating *M. tuberculosis* genetic diversity. Nevertheless, the various findings support the idea of a longstanding host-pathogen co-evolution and the hypothesis that TB spread together with the human out-of-Africa migration [2,12,13]. To investigate the *M. tuberculosis* genetic diversity within and between different populations could therefore give important insights into the dynamics of TB disease and might help to inform national TB programs to develop better control strategies.

Papua New Guinea (PNG) is a high TB burden country with an estimated TB incidence rate of 348/100’000 in 2012 and its proportion of multidrug resistance (MDR) estimated at 4.9% in new cases is higher than the estimated global average of 3.6% [14,15]. Publications on drug resistant TB in PNG are rare, and previous data were mainly derived from patients from Western Province, diagnosed in Australia [16-19]. Gilpin *et al.* reported 25% MDR-TB among patients from Western Province diagnosed between 2000 and 2006 [17]. Only two publications on DR data from other provinces exist: Ballif *et al.* found that 5.2% of tested isolates of adult patients from Madang were MDR-TB [20], and a recently published study from Kikori in Gulf Province of PNG reported 9% of suspected MDR-TB (based on Xpert® MTB/RIF) in the investigated population [21].

PNG harbours a vast human genetic diversity and has been isolated from the outside world for a long time. PNG was populated through several waves of human migrations and people living in the Highlands represent the oldest population from the first migration wave about 50,000 years ago [22,23]. The highlands region has only been ‘discovered’ in the 1930s [24] and was sparsely populated at the time. Hence it would be expected that evolutionary ‘ancient’ lineages of *M. tuberculosis* (e.g. Lineage 1 [25])*,* proposed to be adapted to low density populations [26], would be found there, whilst the ‘modern’ lineages such as Lineage 2 and Lineage 4 would be expected at the highly populated coastal regions. However, there is limited information on the TB situation available in PNG. Since various lineages have been found to differ in their prevalence between different regions of the same country (e.g. in Indonesia [27] or Taiwan [28]) it is important to also identify the circulating strains in various communities in PNG. To our knowledge, apart from a previous study conducted in Madang province [29], another study where isolates from patients from Western Province were analysed [17] and a very recent study from Gulf Province [21], no other data on the *M. tuberculosis* population structure from PNG have been published.

The aim of the present study was to obtain baseline data on the level of drug resistance and the genotypic diversity of circulating *M. tuberculosis* in selected sites of three provinces of PNG (Goroka in Eastern Highlands Province, Alotau in Milne Bay Province and Madang in Madang Province) and to investigate the differences between these sites.

## Results and discussion

### Study population characteristics

**Figure 1:**
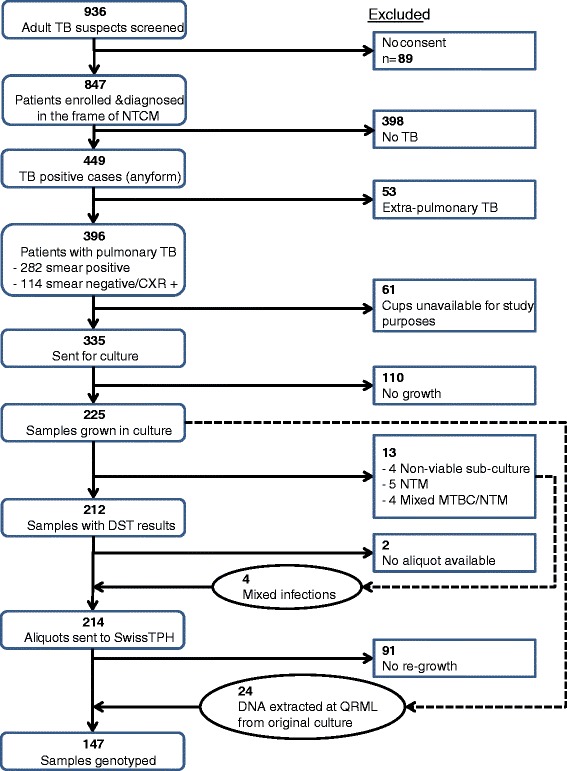
**Flow chart of cohort sample size development from screening to genotyping.**

A total of 449 patients enrolled into our study were diagnosed with TB of any type. From 396 of these patients sputum samples could be collected and of these 335 (74.6%) samples were available for study purposes (see Figure 1). From 212 (63.3%) of the available samples *M. tuberculosis* were successfully grown in culture and drug susceptibility testing (DST) was performed. Details of population characteristics per study site are described in Table 1.

**Table 1:** **Characteristics of study population for each study site**

		Drug susceptibility testing result available*				**Total TB positive (any type)**
**Characteristics**		**Goroka n = 56 n (%)**	**Alotau n = 66 n (%)**	**Madang n = 90 n (%)**	**Total n = 212 n (%)**	**All sites n = 449 n(%)**
**Sex**						
	Male	32 (57.1)	28 (42.4)	43 (47.8)	103 (48.6)	221 (49.22)
	Female	24 (42.9)	38 (57.6)	47 (52.2)	109 (51.4)	228 (50.78)
**Median age [IQR]**		30 [25-40]	29 [24-42]	30 [24-40]	30 [24-40]	30 [25-42]
**Age group**						
	15-24:	13 (23.2)	19 (28.8)	27 (30.0)	59 (27.8)	110 (24.5)
	25-34:	19 (33.9)	23 (34.9)	31 (34.4)	73 (34.4)	157 (35.0)
	35-44:	12 (21.5)	9 (13.6)	16 (17.8)	37 (17.5)	76 (16.9)
	45-54:	7 (12.5)	8 (12.1)	9 (10.0)	24 (11.3)	51 (11.4)
	55-64:	4 (7.1)	4 (6.1)	5 (5.6)	13 (6.1)	40 (8.9)
	>64:	0	2 (3.0)	1 (1.1)	3 (1.4)	11 (2.4)
	missing:	1 (1.8)	1 (1.5)	1 (1.1)	3 (1.4)	4 (0.9)
**Smear result**						
	Smear positive	55 (98.2)	62 (93.9)	90 (100)	207 (97.6)	280 (62.4)
	Smear negative	1 (1.8)	4 (6.1)	0	5 (2.4)	116 (25.8)
**HIV**						
	HIV+	12 (21.4)	1 (1.5)	3 (3.3)	16 (7.5)	36 (8.0)
	HIV-	23 (41.1)	12 (18.2)	43 (47.8)	78 (36.8)	140 (31.2)
	HIV unknown	21 (37.5)	53 (80.3)	44 (48.9)	118 (55.7)	273 (60.8)
**Region of origin**						
	Highlands	25 (44.6)	0	3 (3.3)	28 (13.2)	71 (15.8)
	Coast	4 (7.2)	23 (34.9)	84 (93.3)	111 (52.3)	171 (38.1)
	Other country	0	0	1 (1.1)	1 (0.5)	1 (0.2)
	Unknown	27 (48.2)	43 (65.1)	2 (2.2)	72 (34.0)	206 (45.9)
**History of TB treatment**						
	No	50 (89.3)	42 (63.6)	75 (83.3)	167 (78.8)	355 (79.1)
	Yes	2 (3.6)	5 (7.6)	11 (12.2)	18 (8.5)	50 (11.4)
	Unknown	4 (7.1)	19 (28.8)	4 (4.5)	27 (12.7)	44 (9.8)

*Data of 212 samples for which drug susceptibility was tested compared to 449 enrolled patients diagnosed with any type of TB. IQR = interquartile range.

TB positivity peaked in the age group of 15 to 34 years, which is consistent with country wide data [30]. And 79.1% (355/449) of patients clinically diagnosed with TB had no history of the disease indicating ongoing transmission. However, these data cannot be used to extrapolate for the whole country and further investigations are required to analyse the transmission dynamics of the disease in PNG.

Figure 1 depicts the flow of all isolates collected. Of samples sent for culture 67.2% (225/335) could be recovered showing a positive correlation between initial diagnostic bacterial count and culture success (likelihood ratio χ2 = 140.6, df = 4, p < 0.001). The reduced culture success might be likely due to the long distance transport of samples between laboratories that was experienced with samples from other studies.

### Genotyping

**Figure 2:**
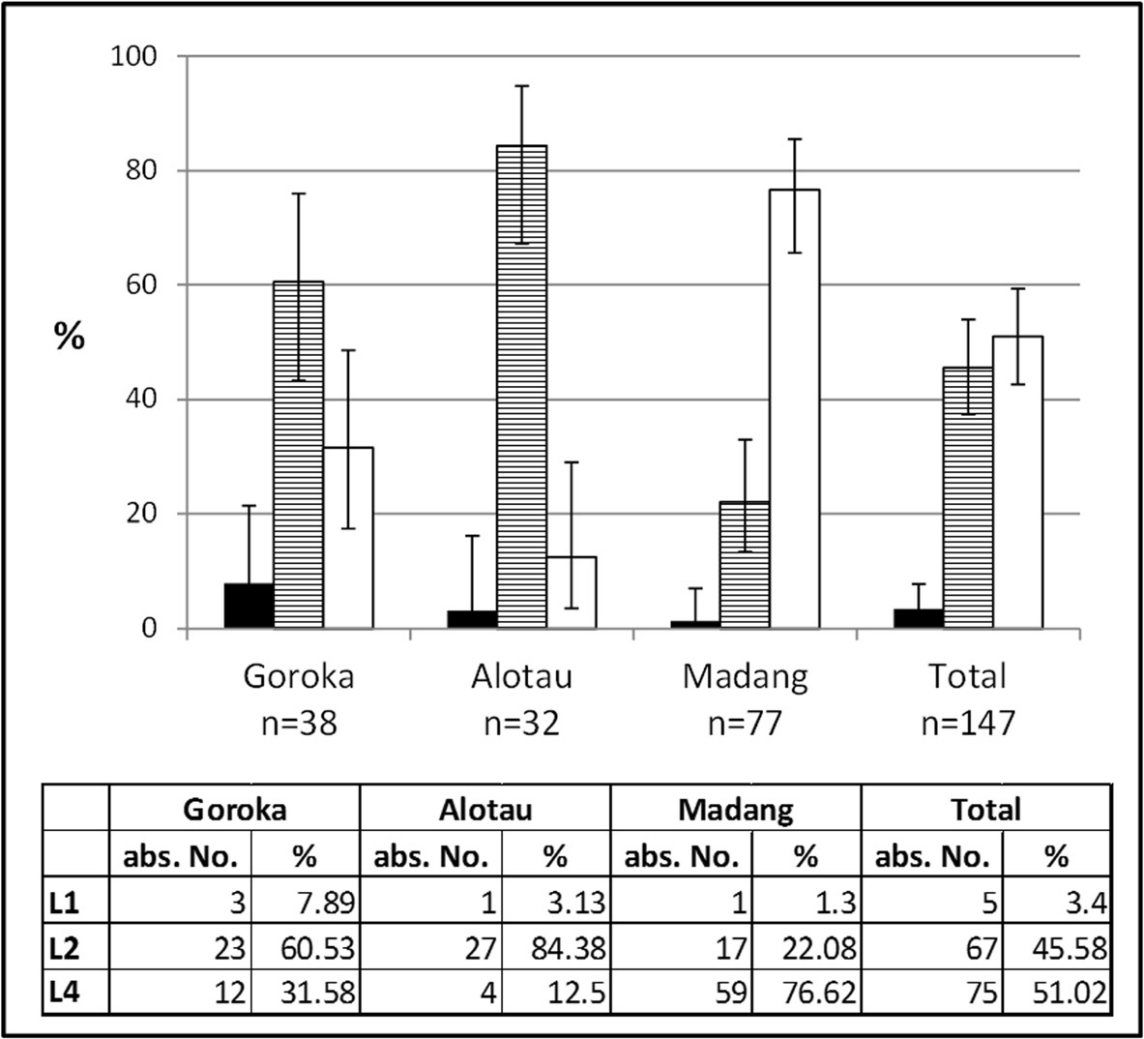
**Mycobacterium tuberculosis lineage composition for each study site.** Black bars = lineage 1 (Indo-Oceanic); dashed bars = lineage 2 (East-Asian); white bars = lineage 4 (Euro-American).

Genotyping of MTBC could successfully be conducted of 147/212 (69%) samples. With a TaqMan Real-time PCR assay [31] three of seven worldwide reported MTBC lineages [3,12], were detected in all three study sites. Overall, Lineage 4 was the most prevalent lineage with 75/147 isolates (51.0%), followed by Lineage 2 with 67/147 isolates (45.6%), whilst Lineage 1 was rare with 5/147 isolates (3.4%). This composition of circulating lineages was similar as previously described for Madang [29] and other parts of PNG [17,21,32] and reflects the reduced MTBC diversity within PNG compared to other countries of the region, e.g. Indonesia [27] or New Zealand [33] where Lineage 3 strains were also found. When lineage compositions (shown in Figure 2) were compared between the study sites, statistically significant differences were observed between all three sites (Fisher’s exact test (p < 0.001)). Lineage 1 was generally rare in all three sites. Lineage 4 was the most abundant lineage in Madang (76.6%) similar to what has previously been described [29]. In contrast, in Alotau Lineage 2 was the dominant lineage (84.4%). In Goroka, a trend towards a higher prevalence of Lineage 2 (60.5%) was found but was not as high as in Alotau. However, it cannot be ruled out that the low proportion of Lineage 1 in our cohort is due to the strain genetic background itself, e.g. exhibiting reduced transmissibility [34] and a lower growth rate in macrophages compared to Lineage 2 and Lineage 4 [11] potentially leading to a decreased culture recovery of Lineage 1.

**Figure 3:**
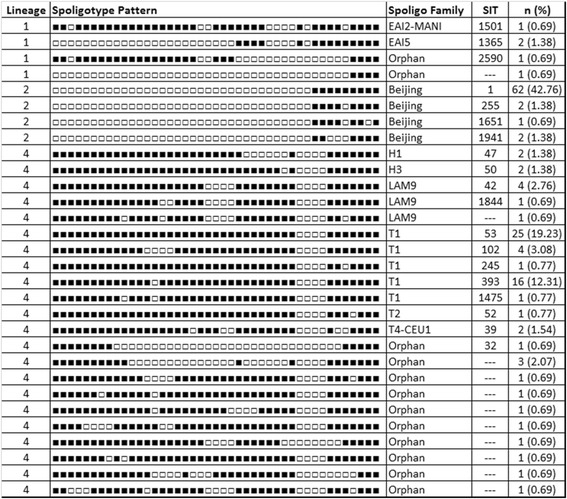
**Spoligotypes for the observed lineages (n = 145).** SIT = shared international type.

We further performed spoligotyping, and the spoligotyping pattern with the corresponding families and frequencies are shown in Figure 3. Thirty different spoligotyping patterns belonged to nine different families, including 14 orphans with 12 different spoligotyping patterns. Orphans were strains with no matching entry in the SITVIT database and are therefore considered as undefined. Whether these strains represent PNG specific *M. tuberculosis* strains remains to be confirmed using other methods.

One of the Lineage 1 strains from Goroka exhibits a special spoligotyping pattern with only spacers 40–43 being present (sample No. 4 in Figure 3). That spoligotyping pattern has been confirmed with in silico spoligotyping using KvarQ [35]. Per standard definition, this would define that strain as belonging to the Beijing family of Lineage 2 [36,37]. However, SNP-typing assigned this strain to Lineage 1, therefore representing a ‘pseudo-Beijing’ strain with convergent evolution leading to an independent deletion event in the direct repeat locus of MTBC, similar to what has already been described for strains of Lineage 3 [38].

All Lineage 4 samples belonging to the LAM spoligotyping family were exclusively found in Madang, also reflecting the different *M. tuberculosis* lineage composition in the spoligotyping data. All Lineage 2 samples belonged to the Beijing family and were subsequently subtyped into three different monophyletic groups which are sublineages defined by the presence or absence of specific regions of difference (RD) [36]. Over all three sites, one sample of 67 Beijing strains belonged to sublineage 1, here defined as having no deletion of RD181, RD150 or RD142. One sample belonged to sublineage 2 with RD181 deleted but RD150 and RD142 being present, but the majority of Beijing strains (63/65), including all Beijing strains from Alotau, belonged to sublineage 3 with RD180 and RD150 deleted. None of the samples had a deletion for RD142. Sublineage 3 is usually rare, hence the high frequency in our sample is surprising. Several studies have shown that sublineage 3 has rapidly spread in Cape Town, South Africa during the last decade, probably because of a founder effect and/or adaptation to the local host-population [39-41]. Due to the lack of longitudinal data, it is not possible to draw any conclusion about the time of introduction or the duration of spread of this subtype in PNG. However, except for two outliers, our subtyping data based on RDs implies a single introduction of the Beijing strain and a subsequent clonal expansion through PNG. Starting with this observation, it would be important to follow the prevalence of this lineage in Madang and in the country as a whole to monitor a potential increase of Beijing strains in the future.

The reasons for the observed differences in lineage frequencies in the three study sites surveyed remain speculative. These sites differ in several aspects, e.g. Goroka in the Highlands had been much longer isolated from the outside world for much longer than the two coastal sites. The population in the Highlands are believed to be descendants of the oldest human migration wave that populated the country [22], however, no statistically significant difference in the prevalence of Lineage 1 in Goroka could be detected.

Other factors might also influence the lineage distribution. Goroka and Madang are connected to the country through the highlands highway, whilst Madang and Alotau have direct access to the island of PNG and beyond. Similarly, the host genetic background and environmental or circumstantial factors such as co-infections or age could well influence the *M. tuberculosis* lineage distribution. We therefore performed univariate and multivariate logistic regressions to test whether infection with a Lineage 2 strain (equals an infection with Beijing strain) was associated with other factors such as DR, HIV status, age and gender (Table 2). Samples not belonging to Lineage 2 were pooled for the analysis, consisting of five samples of Lineage 1 and 75 samples of Lineage 4. Univariate logistic regression confirmed the differences in lineage distribution between the study sites. These differences remained significant also when correcting for possible confounders in a multivariate regression.

**Table 2:** **Univariate and multivariate logistic regressions for the risk of an infection with a Beijing strain of lineage 2**

**Explanatory variable**	**Lineage 2 n = 67 (45.58%)**	**Non-lineage 2 n = 80 (54.42%)**	**Univariate regression: associations with** ***M. tuberculosis*** **lineage 2**		**Multivariate regression: associations with** ***M. tuberculosis*** **lineage 2**	
			**Crude OR (95% CI)**	**p-value**	**Adj. OR (95% CI)**	**p-value**
**Study site n (%)**						
Goroka*	23 (34.33)	15 (18.75)				
Alotau	27 (40.30)	5 (6.25)	3.5 (1.11 - 11.18)	0.033	3.3 (1.0 - 10.8)	0.047
Madang	17 (25.37)	0 (75.00)	0.2 (0.08 - 0.43)	<0.001	0.2 (0.1 - 0.4)	<0.001
**Sex, n (%)**	31 (46.27)	37 (46.25)	1 (0.52 - 1.91)	0.99		
Men*	36 (53.73)	43 (53.75)				
Women						
**Age - median years [IQR]**	30 [25-42]	29 [22-40]	1 [0.99 - 1.05]	0.13	1.0 [0.98 - 1.1]	0.218
**HIV status**						
Negative*	19 (63.33)	44 (95.65)	12.74 (2.57 -			
Positive	11 (36.67)	2 (4.35)	63.07)	0.002		
**TB treatment history, n (%)**						
No*	55 (91.67)	66 (86.84)				
Yes	15 (11.03)	10 (13.16)	0.60 (0.19 - 1.86)	0.38		
**Drug resistance (any type)**						
No*	54 (83.08)	72 (92.31)				
Yes	11 (16.92)	6 (7.69)	2.44 (0.85 - 7.02)	0.097	2.5 (0.7 - 8.5)	0.153

*reference category.

IQR = interquartile range; OR = odds ratio. A p-value <0.05 was considered statistically significant.

Co-infection with HIV has several times been shown to be associated with infection with strains of the Beijing type [42,43]. We also found a significant association between HIV positivity and infection with a Lineage 2 strain (p = 0.002), but due to sample size multivariate logistic regression could not be performed. However, HIV prevalence in PNG is still comparably low, in particular in Alotau (2.1% in Milne Bay Province (Alotau), 5.2% in Eastern Highlands Province (Goroka) and 2.8% in Madang Province) [44] and is therefore unlikely to play a major role in the distribution of lineages.

It is important to note that all differences are between the frequencies of Lineage 2 and Lineage 4, whilst Lineage 1 played no major role, although we would have expected more ancient lineages (Lineage 1) in the highlands. Lineage 4 and Lineage 2 are not only the most prevalent *M. tuberculosis* lineages in PNG, but are also predominating globally [3]. Thus, our findings could support the notion of Hershberg and colleagues [2] that the lineage distribution between and within countries might become homogenized with increasing migration. In other words, more virulent strains such as Lineage 4 and Lineage 2 might slowly replace the ancient lineages as has already been observed in Cameroon [45] and might have started in PNG.

### Drug resistance

In total 23/212 (10.8%) samples were resistant to at least one of the drugs tested. The details of the DR patterns and their frequency in each study site are shown in Table 3. With 6.6% (14/212) the proportion of isolates resistant (mono-resistant or poly-resistant) to Streptomycin (STR) was the highest in our cohort. High STR resistance in PNG is well known and has been found in previous studies [18-20]. In the past STR has been used frequently as single drug to treat TB but also urinary tract and *Klebsiella* infections [46] which might have led to drug resistance. Drug resistance of any type and MDR frequencies were highest in Alotau with 4.6% of MDR cases, but monoresistance was observed more often in Goroka (8.9%) and Madang (6.7%) compared to Alotau (4.6%). However, none of these differences were statistically significant (p = 0.960).

**Table 3:** **Observed phenotypic drug resistance per study site**

		**GKA n = (%)**	**ALO n = (%)**	**MAG n = (%)**	**TOTAL n = (%)**
Pan-susceptible		50 (89.3)	58 (87.8)	81 (90.0)	189 (89.2)
Monoresistant		5 (8.9)	3 (4.6)	6 (6.7)	14 (6.6)
	STR	1	3	3	7
	INH (0.1 mg/L)	2	0	1	3
	INH (0.4 mg/L)	0	0	1	1
	RMP	2	0	1	3
Polyresistant		0	2 (3.0)	1 (1.1)	3 (1.4)
	STR + INH	0	1	1	2
	STR + RMP	0	1	0	1
MDR		1 (1.8)	3 (4.6)	2 (2.2)	6 (2.8)
	INH + RMP	0	0	1	1
	STR + INH + RMP + EMB	1	0	0	1
	STR + INH + RMP	0	2	0	2
	STR + INH + RMP + ETH	0	1	0	1
	INH + RMP + PZA + ETH	0	0	1	1

GKA = Goroka, ALO = Alotau, MAG = Madang, STR = Streptomycin, INH = Isoniazid, RMP = Rifampicin, EMB = Ethambutol, ETH = Ethionamide, PZA = Pyrazinamide.

**Figure 4:**
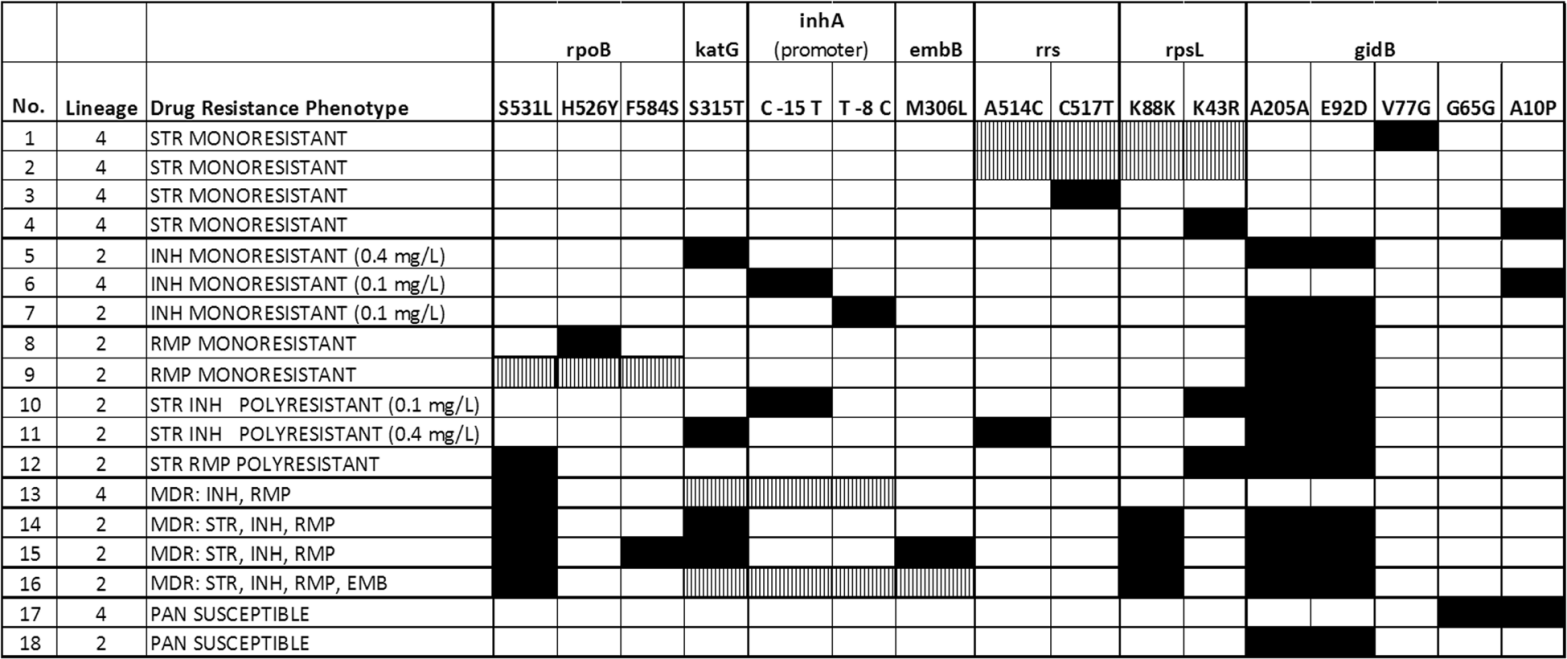
**Overview of genes/gene regions sequenced and mutations observed.** No mutations were found in *ahpC, pncA* and *gyrA*. Black squares: mutation detected at indicated position; white squares: no mutation found at indicated position; dotted squares: no mutation found at any of the indicated positions, which are the most commonly known sites of mutation for the detected phenotypic drug resistance patterns. All positions indicate the amino acid change at the codon position, except for *rrs* gene where nucleotide position and change is indicated.

In order to determine drug resistance mechanisms of 16 phenotypically drug resistant isolates for which DNA could be obtained (16/23), we determined mutations in ten genes known to be associated with resistance. Two pan-susceptible strains of patients with late sputum conversion were also included. Sequence data were analysed and mutations observed are shown in Figure 4.

Streptomycin resistance has been shown to be associated with mutations in the *rrs, rpsL* or *gidB* gene [47,48]. For two STR mono-resistant samples only a mutation in *gidB* was detected (sample 1 in Figure 4) or no mutation was found in any of the analysed genes, respectively (sample 2 in Figure 4). All observed mutations in that gene had also been observed in our previous study in Madang. The sole observation of the synonymous mutation A205G and the non-synonymous mutation A92C in Lineage 2 strains, suggests that these mutations might be lineage specific [20,49] with no mutations occurring in Lineage 4. In three samples, all belonging to Lineage 4, we found the A10P mutation. In contrast to our previous study where the A10P mutation was absent from 21 pan-susceptible samples, we observed this mutation in one of the pan-susceptible samples (sample 17 in Figure 4), probably suggesting that this mutation plays no role in STR resistance. Whether the mutation V77G found here in one STR resistant strain - and yet only described from PNG - is involved in STR resistance [20] remains to be determined.

Ninety-five per cent of the rifampicin (RMP) resistance conferring mutations occur in an 81 bp core region of the so called rifampin resistance determining region (RRDR) [50]. We found only one RMP monoresistant sample (sample 9 in Figure 4) which had no mutation in the 849 bp (including the RRDR) of the *rpoB* gene we amplified, confirming that RMP resistant strains without a typical mutation in the RRDR are not more common in our sample. This is of crucial importance for the PNG DR surveillance being based on Xpert® MTB/RIF (Cepheid) [51] which determines RMP resistance only through detection of mutations in RRDR [52]. However, 26.1% (6/23) of strains were isoniazid (INH) monoresistant or INH/STR polyresistant. INH resistance is a precursor to MDR-TB and is not detected by the Xpert® MTB/RIF, forming an additional challenge for the control of DR TB that should be addressed by the NTP in the future.

All low level INH resistant samples (resistant to a concentration of 0.1 mg/L INH) of our study showed a mutation in the *inhA* promoter region, whereas all high INH resistant samples (0.4 mg/L) showed a mutation at codon 315 of *katG,* including all MDR samples and one of the polyresistant strains with INH and STR resistance (sample 11 in Figure 4). No mutations were detected in the *ahpC* promoter region for any of the samples.

Two MDR samples had no mutation in 850 bp of *katG* sequenced, none in the *ahpC* promoter or the *inhA* promoter. One of these samples was also resistant to ethambutol (EMB) (sample 16 in Figure 4) but had no mutation in the *embB* region including codon 306, which is mutated in up to 68% of clinical EMB resistant strains [48]. For that sample the whole genome sequence was available (*data not shown*) and was used to screen for mutations outside the amplified regions of *katG* or *embB*. Screening revealed a 14 bp deletion at position 2156047 to 2156060 (H37Rv reference, GeneBank AL123456) causing a truncation of *katG* through a frameshift, explaining the phenotypic high INH resistance of that sample. Outside of the sequenced *embB* region the non-synonymous mutation G406S was found which had already been described in several other studies suggesting its role in EMB resistance [53,54]. For the second MDR sample without a *katG* mutation, no sequence data was available and the INH resistance conferring mutation could not be determined. It remains unclear whether a deletion in *katG* or mutations in other genes associated with INH resistance, for example in *kasA* [50] could be responsible for the INH resistance.

No mutation was found in the amplified regions of *pncA* for any of the genotyped isolates*.* However, since for the only sample with phenotypic pyrazinamide resistance (Table 3) no DNA could be obtained, genotyping of that isolate was not possible.

Possible associations with known risk factors for DR were tested using univariate logistic regressions (Table 4). Risk of being infected with a DR strain was 5.5 times higher for patients with a history of TB treatment and 2.4 times higher for patients infected with an Lineage 2 strain although the latter was not statistically significant (p = 0.097). To correct for possible confounders multivariate logistic regression analysis was conducted and the adjusted odds ratio (OR) for risk of being infected with a DR strain with a history of TB treatment decreased from 5.5 to 4.2 but remained significant (p = 0.040). This association highlights the importance of constant access to treatment and compliance for the control of DR TB. Strengthening the DOTS strategy and increasing awareness of TB in the population is crucial also in PNG and needs to be maintained at a high level in order not to delay diagnosis and to prevent the possible spread of TB.

**Table 4:** **Univariate and multivariate logistic regressions for the risk of drug resistance**

**Explanatory variable**	**Pan-susceptible n = 189 (89.15%)**	**Resistant n = 23 (10.85%)**	**Univariate regression: associations with DR**		**Multivariate regression: associations with DR**	
			**Crude OR (95% CI)**	**p-value**	**Adj. OR (95% CI)**	**p-value**
**Study site n (%)**						
Goroka*	50 (26.46)	6 (26.09)				
Alotau	58 (30.69)	8 (34.78)	1.1 (0.4 - 3.5)	0.808		
Madang	81 (42.86)	9 (39.13)	0.9 (0.3 - 2.8)	0.890		
**Sex, n (%)**						
Men*	92 (48.68)	11 (47.83)				
Women	97 (51.32)	12 (52.17)	1.0 (0.4 - 2.5)	0.939		
**Age - median years [IQR]**	29 [24-40]	31 [25-50]	1.0 [1.0 - 1.1]	0.267		
**HIV status**						
Negative*	67 (83.75)	11 (78.57)				
Positive	13 (16.25)	3 (21.43)	1.4 (0.3 - 5.7)	0.636		
**TB treatment history, n (%)**						
No*	155 (92.26)	15 (71.43)				
Yes	13 (7.74)	6 (28.57)	5.5 (1.8 - 16.8)	0.003	4.2 (1.1 - 16.7)	0.040
**Lineage 2**						
No*	72 (57.14)	6 (35.29)				
Yes	54 (42.86)	11 (64.71)	2.44 (0.85 - 7.02)	0.097	3.4 (1.0 - 11.2)	0.041

*reference category.

IQR = interquartile range; DR = drug resistance; OR = odds ratio.

Ballif *et al.* previously found a significant association between an infection with a Lineage 2 strain and drug resistance in a previous study conducted in Madang (p < 0.010, OR = 5.2, CI (95%): 1.8 - 15.1) [29]. Also in our current sample set from Madang a significant association between Lineage 2 strains and drug resistance was found, but only after correction for previous TB treatment (p = 0.041, OR = 3.4, CI (95%): 1.0 – 11.2), probably due to the limited sample size (Table 4). To test whether the two sample sets from Madang (Ballif *et al.* versus Madang samples from current study) differed significantly in the drug resistance data, we compared the results of the drug resistance and Lineage 2 analyses from both sample sets by a χ^2^ test of ORs, but no significant difference was found (χ^2^ = 0.747).

Because of the small sample size we also only found a borderline significant association between multiple drug resistance and Lineage 2 infection (p = 0.058; CI (95%) 0.9 – 68.4; OR = 8): there was an 8 times higher risk of being infected with a polyresistant (resistant to more than one drug but not MDR) or MDR strain when infected with an Lineage 2 strain compared to an infection with a strain of a different lineage (in this case Lineage 4 or Lineage 1).

## Conclusions

The direct comparison of *M tuberculosis* population structures from distinct sites in PNG demonstrated a statistically significant difference between subpopulations. In Madang Lineage 4 was the dominating lineage, whereas Lineage 2 was more frequently detected in Alotau and Goroka. Although the reasons for the observed significant differences of the circulating *M. tuberculosis* strains between study sites are not yet understood, these differences might have a major impact on disease and transmission dynamics in different populations of PNG. Different control strategies for places with a different *M. tuberculosis* lineage composition are not available yet, i.e. the same control strategies apply for all provinces in PNG, namely to detect cases and treat them accordingly. However, by knowing about the increased prevalence of the Beijing type of *M. tuberculosis* in Milne Bay Province, and with the known association between this lineage and drug resistance, monitoring of the latter should especially be scaled up in that province, as it could become a hot spot for drug resistance and MDR TB.

Our data show that a significant number of drug resistant TB infections are present across the country and that MDR TB can already be detected in all three surveyed regions of PNG. Nearly all phenotypical resistances were confirmed by sequence analysis.

No inferences can be made from this study for the whole country because of the small sample size and data being derived from only three major towns. Nevertheless, our findings highlight the importance to monitor drug resistance in PNG, and for making it a high priority for the National TB Control Program.

## Methods

### Study sites and patient characteristics

The study was conducted in three different sites across PNG, one site in each region of PNG: in Madang, Madang Province in the Momase Region; Goroka, Eastern Highlands Province, in the Highlands Region, and in Alotau, Milne Bay Province in the Southern Region of PNG. In Madang, patients were enrolled into the study from November 2010 onwards. In Goroka, patient enrolment started in June 2011 and Alotau was added as a study site in July 2011. In all three study sites enrolment was completed in July 2012. Three consecutive sputum samples were collected from adult TB suspect patients (15 years or older) with chronic productive cough who presented at any department of the provincial hospitals (Modilon Hospital, in Madang; Goroka Provincial Hospital in Goroka; Alotau Provincial Hospital and Gurney Health Centre in Alotau). Questionnaire based interviews were conducted to obtain socio-demographic and behavioural information of each patient. TB was diagnosed by either direct smear light microscopy (Ziehl-Neelson staining), fluorescent microscopy (Morse Stain; TB Fluorescent Stain Kit M, Becton, Dickinson and Company, USA), chest X-ray, clinical examination or a combination of these methods. All TB positive study patients were automatically enrolled into the PNG National TB Program (NTP). Therefore, patient management, i.e. treatment and follow up procedures, was carried out according to the NTP guidelines [51]. For a subset of study patients the HIV status could be obtained from the NTP, which recommends HIV testing of TB positive patients.

### Sample processing and drug susceptibility testing

Sputa were obtained from all tuberculosis patients with pulmonary involvement who were able to produce sputum. Sputum samples were decontaminated according to Petroff’s method [55]. Subsequently, these samples were inoculated into Mycobacterial Growth Indicator Tubes (BACTEC™ MGIT™ 960 system; BD, Franklin Lakes, NJ, USA) and sent to the Queensland Mycobacterium Reference Lab in Brisbane, Australia, for culture and drug susceptibility testing (DST). DST utilising the BACTEC 960 MGIT system was conducted as described previously [29].

### Genotyping of *Mycobacterium tuberculosis*

DNA was extracted from culture either by InstaGene Matrix (Bio-Rad, Hercules, CA, USA) following the manufacturer’s protocol, with one bacterial colony resuspended in 1 ml of dH_2_O as starting material, or by mixing 100 μl of *M. tuberculosis* inoculated into Dubos broth (prepared tubed medium for cultivation of mycobacteria; Becton, Dickinson and Company, Maryland, USA) with 100 μl distilled water and subsequent heat killing at 90°C for 1 hour. The DNA was then used for molecular analyses.

*M. tuberculosis* isolates were classified into the main phylogenetic lineages [12] by a TaqMan real-time PCR assay using single nucleotide polymorphism (SNP) typing [31]. Lineages were further discriminated into families by spoligotyping [56], using a commercial membrane produced by Ocimum Biosolutions Ltd, India (product IM9702). Information on the shared international type (SIT) and the spoligo family were obtained from SITVIT WEB [57]. All Beijing strains were further sub-classified into monophyletic groups based on the presence or absence of the regions of difference (RDs) RD181, RD150 and RD142 as described by Tsolaki *et al*. [36]. PCR conditions and primers were used as previously described by Gagneux *et al.* [3].

### Drug resistance genotyping

Of all phenotypically drug resistant strains for which DNA could be obtained, drug resistance associated regions of the following genes were amplified by PCR and sequenced by Macrogen (The Netherlands): *katG, rpoB, ahpC (promoter), inhA (promoter), gidB, pncA, gyrA, rrs, rpsL, embB*. Additionally, two fully susceptible isolates of patients for whom smear conversion took longer than the usual 2 months were included. Primers and PCR conditions were used as previously described by Ballif *et al.* [20] with the following modifications; for *rpoB* a new set of primers was designed: forward primer 5′AYATCGACCACTTCGGYAACC3′, reverse primer 5′TCCTCGATGACGCCGCTTTCT3′ (Y = C/T). PCR was run with an annealing temperature (T_A_) of 62°C, an elongation time (E_T_) of 60 seconds and 37 cycles, leading to a product length of 849 bp. For the *inhA* promoter and *katG* amplification*,* primers remained the same as published but the T_A_ was increased from 60°C to 65°C and the cycle number from 35 to 39 for *inhA,* and from 64°C to 66°C and from 35 to 40 cycles for *katG*.

### Statistical analysis

Statistical analysis was carried out with Stata 12.1 (StataCorp, College Station, TX, USA). Differences between study sites were assessed by cross-tabulation and significance testing using Fisher’s exact and χ^2^ testing. P-values <0.05 were considered statistically significant. Univariate logistic regressions were performed to assess associations of drug resistance or Lineage 2 infection with known potential predictors or confounders. Independent variables with a significance level of p < 0.2 in the univariate analysis and a plausible causal link where further analysed in a multivariate logistic regression. Model selection for multivariate regressions was based on the AIC-criterion. The comparison of two different sample sets from the same study site was done with a χ2 test of odds ratios with χ2 > 3.84 considered to show a statistically significant difference (5% level).

### Ethical approval

Ethical approval for this study was granted by the PNG IMR Institutional Review Board (IRB No. 0913) and the PNG Medical Research Advisory Council (MRAC No. 10.02). The Ethik-Kommission beider Basel (EKBB) has been informed and had approved the study. Written informed consent was obtained from all study participants.
